# LAMA: automated image analysis for the developmental phenotyping of mouse embryos

**DOI:** 10.1242/dev.192955

**Published:** 2021-03-24

**Authors:** Neil R. Horner, Shanmugasundaram Venkataraman, Chris Armit, Ramón Casero, James M. Brown, Michael D. Wong, Matthijs C. van Eede, R. Mark Henkelman, Sara Johnson, Lydia Teboul, Sara Wells, Steve D. Brown, Henrik Westerberg, Ann-Marie Mallon

**Affiliations:** 1 Medical Research Council Harwell Institute, Harwell OX11 0RD, UK; 2 MRC Human Genetics Unit, MRC Institute of Genetics and Molecular Medicine (IGMM), University of Edinburgh, Edinburgh EH4 2XU, UK; 3 BGI Hong Kong, 26/F, Kings Wing Plaza 2, 1 On Kwan Street, Shek Mun, New Territories, Hong Kong; 4 School of Computer Science, University of Lincoln, Lincoln LN6 7TS; 5 Mouse Imaging Centre, Hospital for Sick Children, Toronto, Ontario M5T 3H7, Canada

**Keywords:** Automated, Computational, Embryo, Micro-CT, Mouse, Phenotyping

## Abstract

Advanced 3D imaging modalities, such as micro-computed tomography (micro-CT), have been incorporated into the high-throughput embryo pipeline of the International Mouse Phenotyping Consortium (IMPC). This project generates large volumes of raw data that cannot be immediately exploited without significant resources of personnel and expertise. Thus, rapid automated annotation is crucial to ensure that 3D imaging data can be integrated with other multi-dimensional phenotyping data. We present an automated computational mouse embryo phenotyping pipeline that harnesses the large amount of wild-type control data available in the IMPC embryo pipeline in order to address issues of low mutant sample number as well as incomplete penetrance and variable expressivity. We also investigate the effect of developmental substage on automated phenotyping results. Designed primarily for developmental biologists, our software performs image pre-processing, registration, statistical analysis and segmentation of embryo images. We also present a novel anatomical E14.5 embryo atlas average and, using it with LAMA, show that we can uncover known and novel dysmorphology from two IMPC knockout lines.

## Abstract (precis)

**Summary:** Introducing an easy-to-use automated anatomical phenotyping pipeline for mouse embryos along with a highly-detailed anatomical E14.5 atlas.

## INTRODUCTION

A major goal in biomedical research is to assign functional roles to all genes in order to shed light on disease mechanisms, and to identify disease-associated genes and novel drug targets. However, almost two decades since the human and mouse draft genomes were published (Lander et al., 2001; Waterston et al., 2002), the proportion of genes in the dark genome, defined as those having minimal gene function or disease-association annotations, remains high at over 30% (Oprea, 2019). The International Mouse Phenotyping Consortium (IMPC) is a high-throughput functional genomics project tasked with generating a genome-wide catalogue of gene function by phenotyping gene knockouts on a uniform genetic background (Brown and Moore, 2012; Lloyd et al., 2020). Phenotype annotations for over 7000 genes are currently available on the IMPC web portal (mousephenotype.org), data that have already contributed to the identification of many novel candidate disease genes and new mouse models of human disease (Cacheiro et al., 2020; Meehan et al., 2017; Bowl et al., 2017; Moore et al., 2018).

Postnatal lethality or subviability is observed in approximately one-third of knockout mouse lines from both the IMPC (Dickinson et al., 2016) and its precursor EUMODIC (Hrabě de Angelis et al., 2015). These classes of genes provide important insights into developmental processes and disorders. The IMPC seeks to phenotype these classes of gene through the embryo phenotyping pipeline at key embryonic developmental stages (E14.5, E15.5 and E18.5) via the generation and analysis of high resolution, whole embryo, 3D images (Adams et al., 2013). There are currently several thousand 3D images across hundreds of mutant lines at the IMPC, which will be impractical to manually annotate by domain experts as this can take several hours per image (Wilson et al., 2016). Therefore, a high-throughput, generalisable analysis method is needed to extract phenotype associations from these data. An automated method will also mitigate any user bias that may negatively affect reproducibility.

One approach is to automate phenotypic annotation using voxel-based morphometry (VBM) in which 3D images are spatially aligned to allow voxel intensities or deformation fields to be statistically analysed in order to identify morphological differences. This approach, originally developed for human brain MRI images (Wright et al., 1995), proved to be suitable for the analysis of MRI whole-embryo images of E15.5 mice (15.5 days post coitum) which was able to identify morphological differences between wild-type inbred strains (Zamyadi et al., 2010). This work was expanded to the analysis of micro-CT images of mutant E15.5 embryos, showing that known dysmorphology could also be identified using this approach (Wong et al., 2014).

Statistical parametric heatmaps obtained from VBM analysis can be overlaid onto the registered images in order to highlight regions of dysmorphology, facilitating manual annotation by an expert anatomist. But in order to automate the assignment of anatomical phenotypes, an atlas is required. An atlas consists of an image volume where visible anatomical structures have been manually delineated and identified, which enables the automatic calculation of organ volume when combined with VBM. An atlas can be derived from a single reference image, but a population average consensus reference image formed from spatially normalising multiple specimens provides increased signal to noise and contrast (Holmes et al., 1998) making it easier to segment. The population average also provides a less biased registration target from which to propagate anatomical labels. Mouse embryo population average models and associated atlases have been developed for the E15.5 developmental stage using MRI (Cleary et al., 2011) and micro-CT (Wong et al., 2012) where six and 48 anatomical structures were segmented, respectively.

Sample sizes of both mutant and wild-type embryos are important considerations in any phenotyping experiment. Wong et al. (2012, 2014) proposed using eight wild types and eight mutants in their pipeline; however, knockout lines submitted to the IMPC often have much lower sample sizes. Despite the fact that knockout lines are generated from isogenic inbred mice, they frequently exhibit incomplete penetrance and variable expressivity of phenotypes (Wilson et al., 2017; Dickinson et al., 2016), reducing the statistical power to uncover gene-level phenotypes. One solution to this problem is to increase the number of control specimens in order to increase statistical power. However, there are two features of the previous method (Wong et al., 2014) that place restrictions on increasing sample numbers. First, the groupwise registration steps increase computational cost exponentially with increased sample number, and second, the wild-type controls must be registered along with specimens from each mutant line into a unique coordinate space and so cannot be reused for the analysis of other genes.

Another complicating factor in the study of mouse embryos is the presence of inter- and intra-litter variability in developmental stage and in associated morphological differences, which are frequently observed even with inbred wild-type mice (Miyake et al., 1996; Geyer et al., 2017). Indeed, fertilisation time is only estimated by the presence of vaginal plugs, and therefore may vary within a litter along with rate of development. Therefore, embryos harvested from a litter will present a range of developmental sub-stages that can span up to several hours, which corresponds to significant morphological differences in embryos. Therefore, to avoid spurious annotations or masking of real dysmorphology, it is essential to control for developmental stage. These issues of both partial penetrance and of developmental stage variability have yet to be studied in the context of automated phenotyping using 3D images.

In this article, we introduce a new automated phenotyping pipeline (LAMA) that is designed to address the issues arising from the high-throughput analysis of 3D mouse embryo data from the IMPC pipeline. One of the main differences in LAMA when compared with previous work (Wong et al., 2014) is the use of a registration strategy where all baseline and mutant specimens are registered directly towards a population average target in a pairwise manner with no groupwise registrations. This allows a large increase in the number of wild-type controls that can be used when analysing mutant lines, which greatly increases statistical power. Using E14.5 embryo images, a timepoint yet to be subject to this form of automated annotation, we show that with this increase in power, LAMA is able to identify sex differences using a low sample number, and that previously known and novel phenotypes can be uncovered from two knockout lines. Importantly, in one of these lines, LAMA is able to assign known phenotypes to individual specimens, which has not been shown previously for the automated analysis of mouse embryos. We report the results of the effect of developmental substage on automated analysis and include an updated statistical model to account for this. To accompany the pipeline, we present a novel, highly detailed anatomical atlas of an E14.5 population average with 184 labels and associated Mouse Developmental Anatomy Ontology (EMAPA) terms (Hayamizu et al., 2013), which is the most-detailed atlas of a micro-CT population average embryo that is currently available. Finally, we have made LAMA open source and simple to install on all major operating systems and have included tools for preprocessing of data, distributed computing to speed up analysis, and the production of various plots and reports to help users understand the registration process and statistical analyses. These tools, resources and insights presented here will greatly increase the ability to uncover useful phenotype information from embryo imaging data and it is currently being optimised to work with IMPC data from other developmental timepoints.

## RESULTS

### Overview of the LAMA phenotyping pipeline

**Fig. 1.**
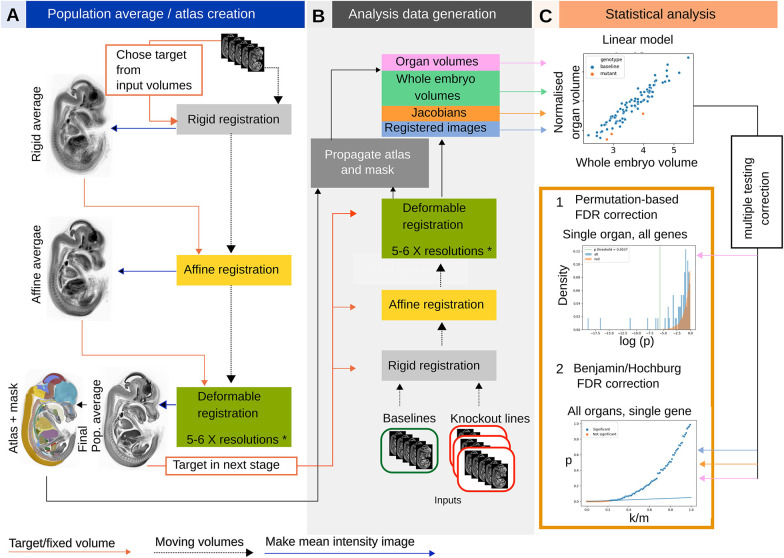
**LAMA pipeline workflow.** (A) Population average construction. A random initial image is used as a target to rigidly align all other images, creating a rigid population average. This is repeated with affine and deformable registration, refining the population average and using it as the target for the next level. (B) Generation of data for phenotype detection. Each test image is registered to the final population average obtained in A, but using the same rigid/affine/B-spline registration levels. (C) Jacobian determinant volumes, registered images and organ volumes are statistically analysed. Top panel: organ volumes (shown here) or voxel values are fitted to a linear model by genotype and whole embryo volume. The resulting genotype effect *P*-values are corrected for multiple testing in one of two ways. (1) permutation-based FDR correction (organ volumes only). The orange histogram shows the null distribution from permuting the wild-type organ volumes and the blue histogram is the alternative distribution derived from testing the organ volume from all mutant lines tested. The vertical green line indicates the calculated *P*-value threshold for this organ, with values lower than this annotated as significant. (2) Benjamini Hochberg FDR correction for voxel-level or organ volume data. k/m=*P*-value rank divided by number of values. The straight blue line indicates the threshold under which values are annotated as significant.

LAMA is a voxel-based morphometry (VBM) approach to automate the detection of anatomical dysmorphology in mouse embryos. It is written in the Python programming language and is distributed as a PyPi package (https://pypi.org/) enabling installation with a single command (see Materials and Methods). It features spatial normalisation of images, using a registration process to iteratively align micro-CT embryo images to a population average image, putting them into the same coordinate space. Internally, it uses elastix (Klein et al., 2010; Shamonin et al., 2014) for multi-resolution 3D image registration. First, LAMA constructs a population average model embryo from wild-type derived images (Fig. 1A) if one does not exist already. This serves as a target image for the subsequent spatial normalisation of the phenotyping images, and as the template for a hand-labelled atlas of mouse organs and anatomical structures (described below) (Fig. 1A). The next step involves spatially normalising each wild-type and mutant image that will be used in the downstream statistical analysis, by registering it towards the population average (Fig. 1B), resulting in morphologically similar images with homologous anatomical structures occupying identical coordinates and existing in the same coordinate space as the atlas.

Each individual specimen image is then automatically segmented by applying the inverse of its registration transformation to the atlas labels (Fig. 1B). Whole-embryo volumes (WEVs) and organ volumes are calculated from these segmentations. Each set of organ segmentations also serves as a specimen-specific visual atlas that can aid in identifying structures in the original inputs and visually detecting segmentation errors. Organ volumes are fitted to a linear model organ volume/WEV∼genotype+WEV. In this model, the organ volumes are first normalised by WEV to control for overall embryo size and the WEV fixed effect accounts for differences due to developmental substage (see the section ‘Developmental substage’). Modelling organ volumes is orders of magnitude less complex than with voxel-based deformation measures (hundreds of data points per line instead of millions), allowing us to employ a more robust permutation-based method for multiple testing correction (see Materials and Methods and Hrabě de Angelis et al., 2015). For this article, we focus on the organ volume analysis as this is linked to the atlas, resulting in automated phenotype calls at the organ level that are more robust and interpretable than those at the individual voxel level. However, we also include statistical parametric heat maps from the voxel-level Jacobian determinants analysis (which indicate local volume shrinkage/expansion during spatial normalisation) for illustrative purposes. The statistical analysis is carried out by combining specimens with the same gene deletion to give gene-level phenotype calls. Each specimen is also analysed individually to give a specimen-level phenotype call, which aims to uncover phenotypes with variability in penetrance or expressivity (see Materials and Methods). During analysis, various plots and information files are generated, including registration metric plots to aid in registration optimisation, organ volume plots and heatmaps that give an overview of the statistical results, and QC report montages showing a snapshot of the automated segmentation results.

### Creation of a novel E14.5 mouse embryo atlas

**Fig. 2.**
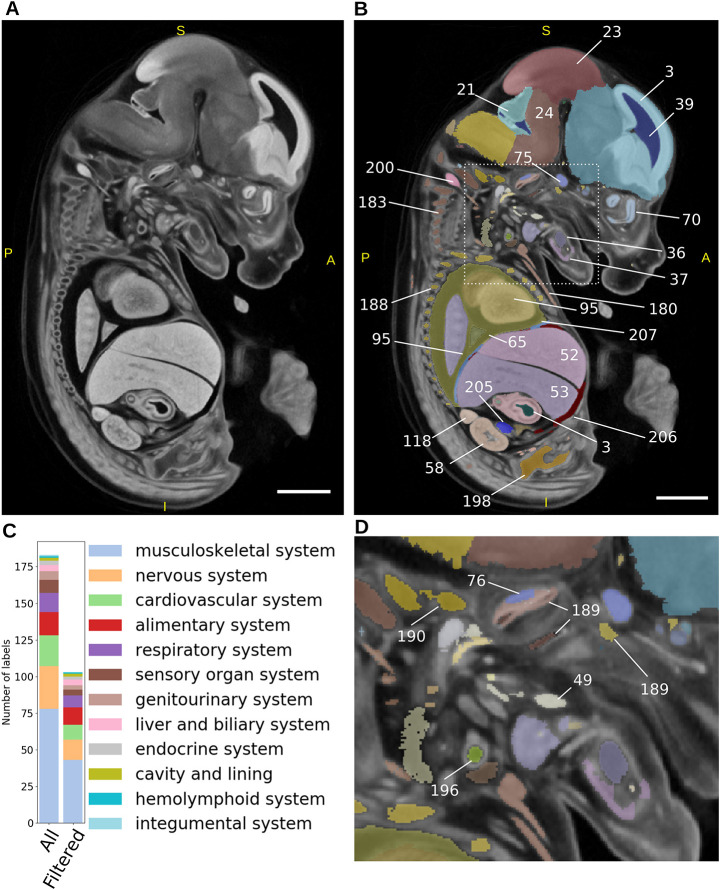
**E14.5 atlas creation**. (A) Sagittal view of the E14.5 population average created from 16 wild-type female and male E14.5 C57BL/6 mice. (B) Sagittal view of the E14.5 atlas consisting of 184 individual structures overlaid on the population average. Only a subset of labels is shown for illustrative purposes. Numbers indicate the labels assigned to segmented organs, which can be looked up in Table S1. (C) Breakdown of labels into corresponding organ systems for the whole atlas (All), and those remaining after filtering small or spindly labels (Filtered) (see Materials and Methods). (D) Expanded region from area outlined in B, highlighting more labels. Scale bars: 1 mm. S, superior; I, inferior; A, anterior; P, posterior.

The E14.5 population average used in this study was created from 16 specimens (eight male and eight female), with a resulting crown-rump length of 9.18 mm (s.d. 0.52 mm) and an isotropic voxel size of 14 μm^3^ (Fig. 2A). The visible organs were segmented using a mixture of manual and semi-automatic segmentation (see Materials and Methods) producing an E14.5 atlas containing 184 unique labels (Fig. 2B,D; Table S1; Movie 1). The 184 labels were mapped to gross anatomy terms from the EMAPA developmental ontology (Hayamizu et al., 2013), where a one to one relationship existed, allowing the automatic integration of the resulting gene-to-phenotype data from LAMA with other data sources that also use this ontology, such as the other IMPC pipelines and MGI (Bult et al., 2019). Through visual inspection of registration results, a number of labels, including blood vessels, nerves and small muscles, were identified as being too small or too thin, leading to difficulty in assessing their registration accuracy. With this in mind, we identified such labels in the atlas (see Materials and Methods) to exclude them from downstream analyses, resulting in a final set of 103 labels that were used in the current analysis (Table S1). These 103 labels were distributed across the majority of the EMAPA high-level organ system terms (Fig. 2C) and range in size from the largest (forebrain at 7.0 mm^3^) to smallest (metatarsals 0.04 mm^3^).

### Developmental substage

**Fig. 3.**
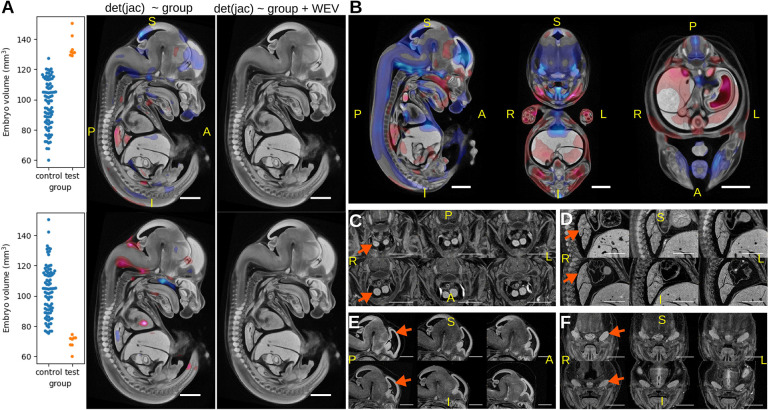
**Effect of E14.5 developmental substage on local volume changes detected by LAMA.** (A) Simulation of the analysis of mutant lines with large (top panels) or small (bottom panels) wild types relabelled as mutants. Genotype effect t-statistics where q values (FDR-corrected *P*-values)<0.05 were overlaid on the E14.5 population average. Left images show the results from the model deformable Jacobians∼genotype, with red voxels highlighting regions that are significantly larger in the test group and blue voxels highlighting regions that are significantly smaller in the test group. Right images show results from the model: deformable Jacobians∼genotype+WEV, where no voxels passed the q<0.05 threshold. (B) Result from fitting 93 wild-type specimens to the linear model deformable Jacobians∼WEV, indicating regions where the normalised organ volume is positively correlated (red) or negatively correlated (blue) to WEV. (C-F) Illustrative examples of differences in organ size relative to embryo volume. Each panel shows three representative wild types from the smallest set (top) and largest set (bottom) of specimens. Images are affinely registered towards the population average to account for overall embryo volume. Arrows indicate relevant anatomy. Thymus (C) and lung (D) show larger relative sizes in larger specimens. Lateral ventricles (E) and trigeminal gland (F) show smaller relative sizes in larger specimens. Scale bars: 1 mm. S, superior; I, inferior; A, anterior; P, posterior; L, left; R, right.

As the developmental substage (DSS) of E14.5 embryos has been shown to be an important consideration when manually phenotyping embryos (Geyer et al., 2017), we did a series of experiments to gauge the effect that DSS has on our automated phenotyping results. To account for overall embryo size, we normalised organ volumes, derived from automatically segmented labels, to whole embryo volume (WEV). Similarly, we removed rigid and affine transformations from the analysis of Jacobian determinants so that the determinants correspond to local deformable transformations (see Materials and Methods) as described previously (Wong et al., 2012) for E15.5 embryos. To assess whether these normalisations are sufficient to account for differences due to DSS, we made two wild-type datasets from the 93 wild-type controls where either the smallest or largest specimens (WEV mean z-score of −1.7 and 1.9, respectively) were relabelled as ‘mutant’. We applied the LAMA Jacobian determinant analysis to each dataset using the linear model deformable Jacobians∼genotype (Fig. 3A), simulating the scenario of two mutant lines containing embryos at early or late E14.5 DSS. Both tests returned significant Jacobian determinant voxels, suggesting that the relabelled wild types had morphological differences that were dependent on the developmental stage of the specimens. WEV was then included as a fixed effect in the following model deformable Jacobians∼genotype+WEV to act as surrogate for DSS. This experiment returned no significant genotype effect voxels, showing that DSS variability can be thus controlled for. To gain a more detailed view on the voxel-level DSS-dependent relative size differences, Jacobian determinants from 93 wild-type specimens we fitted to the linear model deformable Jacobians∼WEV (Fig. 3B), which further highlighted regions that are proportionally larger at later stages (red) or proportionally smaller at later stages (blue). The equivalent test using organ volume analysis organ volume/WEV∼WEV similarly resulted in significant calls for 78/103 labels (Table S2), including organs that were proportionally larger (*n*=58) later in development such as thymus and lung lobes (Fig. 3C,D), and those that were proportionally smaller (*n*=23), including brain ventricles and trigeminal glands (Fig. 3E,F). To summarise, normalising the Jacobian determinants or organ volumes before statistical analysis is not sufficient to account for DSS, and failure to model DSS can lead to false-positive results. Our method resolves this issue by regressing out the DSS effect in the statistical analysis.

### Optimal sample size for phenodeviance testing

**Fig. 4.**
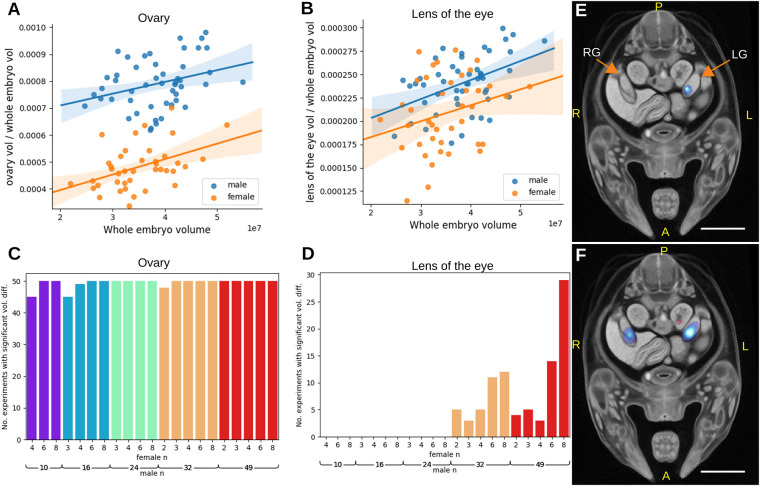
**Identification of sex differences in wild-type E14.5 mice.** (A,B) Plots showing WEV normalised organ volume against WEV for ovary (A) and lens (B). (C,D) A series of statistical tests was carried out with various combinations of male and female wild-type sample size (with females relabelled as ‘mutant’). Each test was repeated 50 times with randomly selected specimens. The values reported are the number of times the gonad volume was reported as significantly different (*P*<organ p threshold) in each set of tests for gonad (C) and lens of the eye (D). (E,F) Example results of Jacobian determinant analysis using eight females and eight males (E), and eight females and 46 males (F). Scale bars: 1 mm. RG, right gonad; LG, left gonad; A, anterior; P, posterior; L, left; R, right.

In order to validate LAMA with a positive control we applied it to wild-type embryos where females were relabelled as ‘mutant’. After removing specimens with indeterminate sex, our test data set contained 49 males and 40 females, in which we expected gross morphological differences between the two sets to be located at the gonads only (see Fig. S1A for example gonad images). These data were fitted to the linear model organ volume/WEV∼sex+WEV and we found that, along with the gonad (FDR-corrected q-value=1.3e^−25^) (Fig. 4A), the lens of the eye unexpectedly also had a significant sex effect (FDR-corrected q-value=0.028) (Fig. 4B; Fig. S1B,C). We next wanted to address the effect of sample size on the sensitivity of phenodeviance detection (in this case the ability to differentiate between male and female gonads and lenses). To do this, the previous experiment was repeated, but with varying numbers of male or female specimens, in this way replicating the effect of testing mutant lines containing various sample numbers and with different baseline control sample numbers (ranging from two to eight females and from 10 to 49 males). Each experiment was repeated 50 times with random specimen selection and permutation-based multiple testing correction (see Materials and Methods for further details). Owing to the large difference between male and female gonad sizes, significant gonad volume differences were identified in almost every replication of each experiment (Fig. 4C), and significant Jacobian determinant voxels were identified within, or close to, the gonad, with significant voxels covering a larger area with increasing male sample size (Fig. 4E,F). For the lens of the eye, significant volume differences were detected only with a male sample size of 32 or over, with the maximum male and female sample size (49 male and eight female) resulting in significant hits in over half of the tests (Fig. 4D). To assess the rate of false-positive detection, any significant organ volumes other than gonad or lens of the eye were classed as false positives. The rate of false positives was found to be well controlled with only 1 out of 103 organs called as significant in more than 1% of tests (epiglottis in 1.6% of all the replications), and with a mean false-positive rate of 0.07% per label. These experiments show that LAMA is able to identify sex-specific differences in wild-type embryos and that even with a low mutant sample size, differences in morphology can be detected, although the detection becomes more reliable as the control sample size is increased.

### Automated identification of developmental phenotypes in E14.5 mice embryos

The initial aim of LAMA was to automatically identify dysmorphology from IMPC-generated data. To demonstrate its effectiveness on IMPC-generated data, we have chosen two exemplar mutant lines that illustrate its use in embryos with multiple dysmorphologies throughout the body and embryos with very specific, localised abnormalities.

**Fig. 5.**
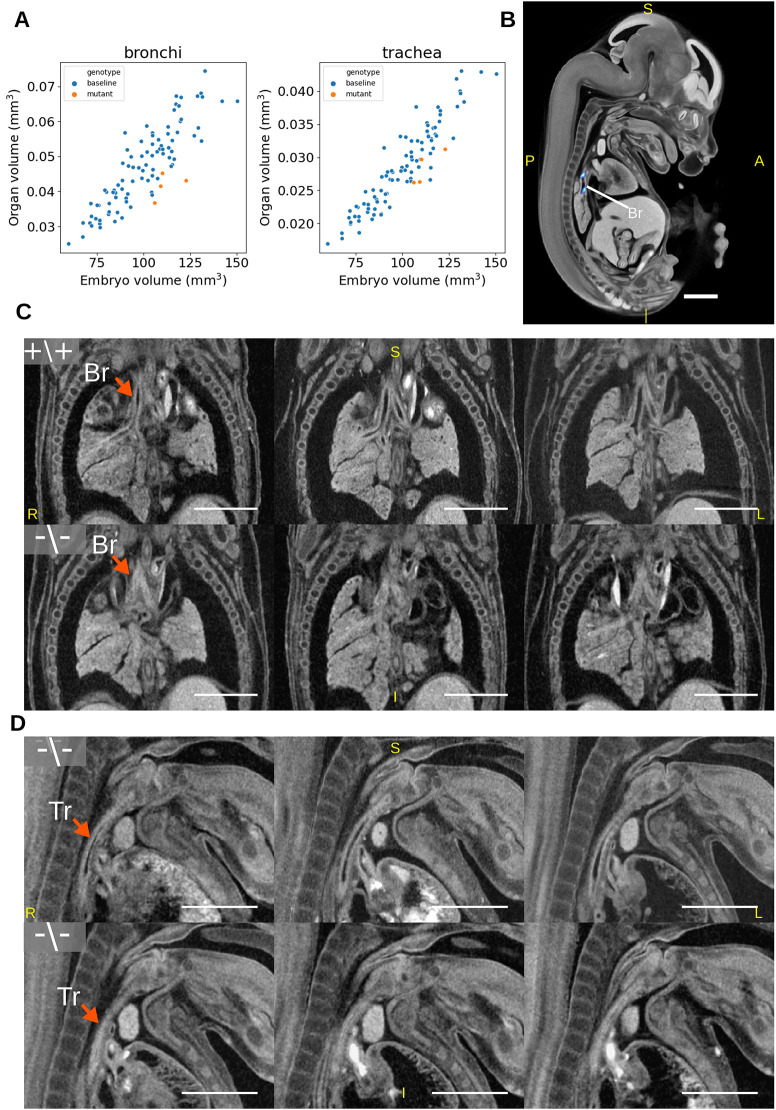
**Automated identification of pulmonary system phenotypes in a *Wfdc2* knockout mouse line.** (A) Organ volume plots of statistically significant organs. (B) Jacobian determinant analysis t-statistics (FDR corrected to q<0.1) overlaid on the E14.5 population average. Blue regions indicate smaller bronchi in the mutants. (C,D) Rigidly aligned sections of *Wfdc2* mutants (bottom) and whole-embryo volume-matched wild-type rigidly aligned specimens (top), with arrows indicating the location of affected organs. (C) Coronal sections highlighting the bronchi. (D) Sagittal sections highlighting the trachea. Scale bars: 1 mm. A, anterior; P, posterior; S, superior; I, inferior; L, left; R, right; Tr, trachea; Br, bronchus.

The first example is *Wfdc2*, which encodes a protease inhibitor protein that is expressed in several tissues during mouse development prior to E14.5 (Lizio et al., 2015), including intestines, lungs and pancreas. *WFDC2* plays a role in cancer development (Bingle et al., 2002; Li et al., 2013) and two articles have recently shown *Wfdc2* homozygous mutant mice display severe pulmonary phenotypes, including collapsed lungs at perinatal day 1.5 (P1.5) (Nakajima et al., 2019), and alveolar abnormalities, dyspnea and reduced blood oxygen saturation at birth (Zhang et al., 2020), but are otherwise anatomically normal. The IMPC viability screen reported that *Wfdc2^−/−^* animals were viable at E18.5 but displayed a partially penetrant preweaning lethality with 5.5% of the alive pups being homozygous for the mutation. Our analysis of four E14.5 *Wfdc2^−/−^* specimens uncovered significantly smaller bronchi and trachea at the gene level (using all four mutants in the analysis) (Fig. 5A) but no significant specimen-level differences (analysing each specimen individually) were observed for this gene. The Jacobian determinant analysis identified two significant regions that largely overlap with one of the bronchi after the FDR-corrected *P*-value threshold was raised to 0.1 (Fig. 5B). This means that, for this gene, the whole organ volume statistics were more sensitive than the Jacobian determinants. In all four mutants, the trachea and bronchus are visibly smaller in diameter (Fig. 5C,D), but otherwise appear normal.

**Fig. 6.**
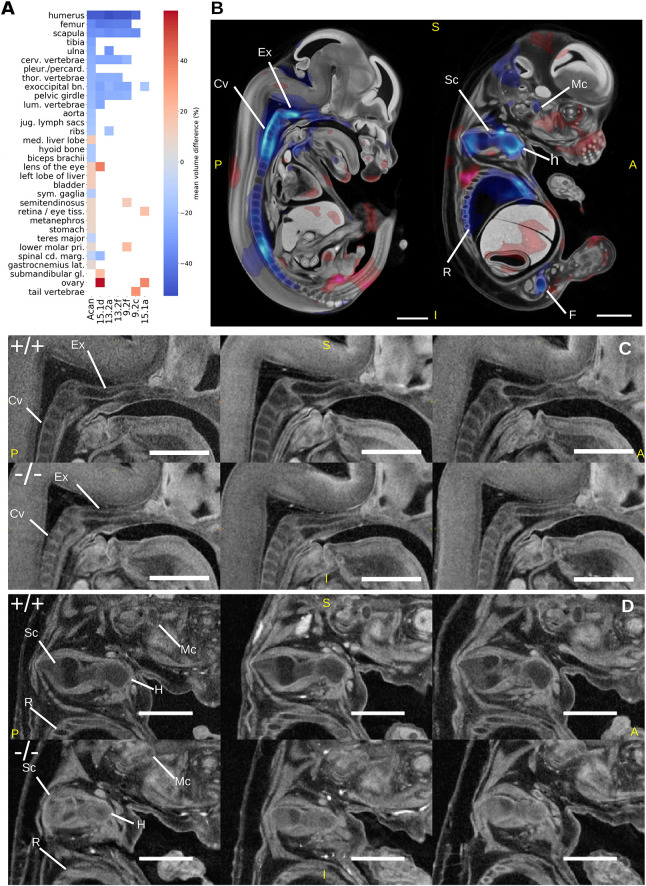
**Analysis of *Acan^−/−^* mutants by LAMA.** (A) Heatmap showing statistically significant organ volume differences at the gene level and of individual specimens. The first column (Acan) are gene-level results and the remaining columns are results from individual specimens. Statistically significant organ volume differences for genotype effect are coloured according to mean normalised volume difference between wild type and mutants. White cells indicate no significant genotype effect. (B) Jacobian determinant t-statistics for genotype effect (FDR corrected to q<0.05) overlaid onto the E14.5 population average. (C,D) Illustrative sagittal slices from rigidly aligned *Acan^−/−^* mutants (bottom) and whole embryo volume-matched wild-type specimens (top) highlighting identified dysmorphology. Scale bars: 1 mm. Mc, Meckels's cartilage; Ex, exoccipital bone; Cv, cervical vertebrae; H, humerus; Sc, scapula; R, ribs; F, femur; A, anterior; P, posterior; S, superior; I, inferior.

*Acan* encodes for the protein aggrecan, which is the primary proteoglycan in articular cartilage, is present in the extracellular matrix of long bone epiphyseal growth plates and is required for normal bone development. *Acan*^−/−^ mice exhibit phenotypes associated mainly with abnormal bone morphology, including long bones, ribs and vertebrae, as well as enlarged liver and pulmonary hypoplasia (Table 1). Human diseases associated with *ACAN* mutations include osteochondritis (Stattin et al., 2010) and skeletal dysplasia (Tompson et al., 2009). The IMPC viability screen reports that *Acan*^−/−^ embryos are viable up to E18.5, but have a completely penetrant preweaning lethality phenotype. Other IMPC-assigned phenotypes include a reduced bone area composition and increased circulating cholesterol levels in adult heterozygous animals. We analysed six E14.5 *Acan^−/−^* specimens with LAMA, identifying 28 statistically significantly gene-level organ volume differences (Fig. 6A; Fig. S2A). Twelve of the significant organs are bones, including all those present in the Mouse Genome Informatics (MGI) annotations for this gene (Table 1). These include smaller cervical vertebrae, scapula, humerus, ribs and exoccipital bones (Fig. 6C,D). Importantly, LAMA was able to assign statistical significance to organ volume differences for 15 of the gene-level annotated organs to individual specimens. Of these organs, most were bones and had the largest mean volume difference, relative to wild-type controls. The specimen-level annotations are similar across all specimens, but with some exceptions, e.g. specimen 15.1a appeared largely unaffected (Fig. 6A). From a total of 42 specimen-level calls, there were four significantly different organ volumes highlighted by the specimen-level analysis that were not present at the gene level, including a tail vertebra annotation (Fig. 6A). The significant Jacobian determinant voxels indicated a smaller Meckel's cartilage in the mutants, which is not identified in previous literature or by our organ volume difference test, but is visibly smaller in these mutants (Fig. 6D). We did not find a significantly smaller lung volume difference for any of the lung lobes that would indicate hypoplasia as previously reported (Houghton et al., 1989), but visual inspection of the lung lobe segmentation labels indicated acceptable registration accuracy (Fig. S2B). The position of the lungs within the thoracic cavity, however, looked altered, possibly owing to changes in the thoracic cavity size. We did not identify the remaining previously reported phenotype of tracheal cartilage morphology either.

**Table 1.**
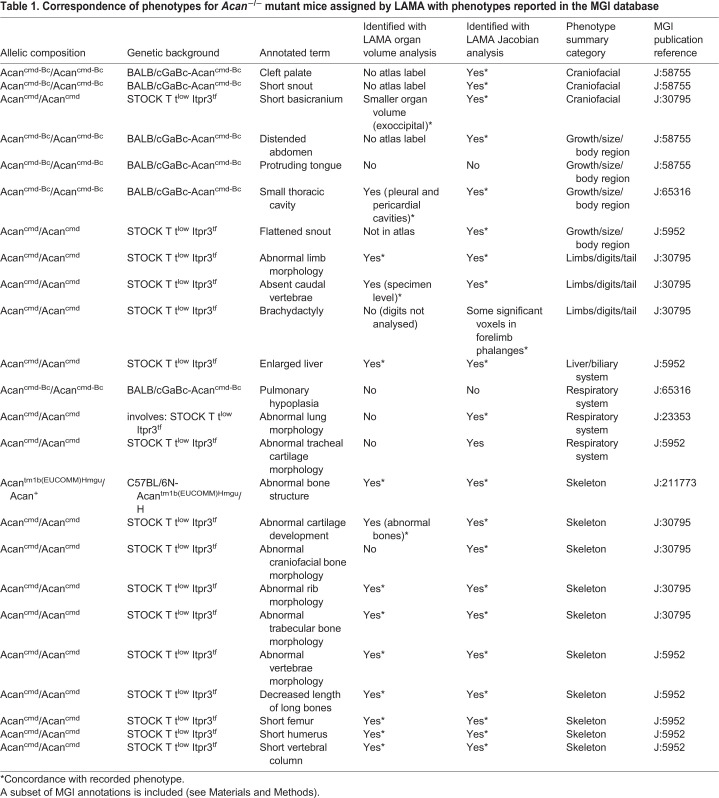
**Correspondence of phenotypes for *Acan*^−/−^ mutant mice assigned by LAMA with phenotypes reported in the MGI database**

## DISCUSSION

In this paper we presented a new automated computational phenotyping pipeline for dysmorphology detection in mutant mouse embryos along with a new E14.5 anatomical atlas. We have undertaken validation of the pipeline and provide insights on the effect of developmental substage and sample size on phenotyping results, as well as showing that LAMA can uncover previously reported and novel phenotypes from E14.5 IMPC knockout mice embryos. This pipeline and atlas will accelerate the automatic analysis of 3D embryo data at this developmental stage within the IMPC (where it is being adapted for other developmental stages), other large scale projects, and smaller challenge-led projects, driving forward the use of disease models in scientific discovery.

The image registration approach in LAMA provides significant advantages for high-throughput phenotyping compared to previous work (Wong et al., 2012, 2014). The major difference is the registration strategy in which all data is registered at once, directly towards a pre-made population average and atlas, which puts all the specimens from all mutant lines and controls into the same coordinate space. This contrasts with the approach of Wong et al. (2014) where specimens from a mutant line and a small number of wild types are registered into a unique coordinate space. With our approach, we were able to dramatically increase the number of wild type control embryos used in the phenotype analysis stage of the pipeline. This is due to the lack of a groupwise registration stage, which decreases computational expense, as well as the ability to reuse wild-type registered specimens in statistical analysis across many mutant lines. A further advantage of our approach is that it facilitates the distributed processing of images as each registration requires only the fixed population average and the moving specimen images.

We have shown that even after normalising organ volumes by whole-embryo volume (WEV), there remains a significant WEV effect, which we interpret as substage-dependent differential organ growth rates. This variation in organ volume after normalising by WEV agrees with findings by Wong et al. (2012), who reported an organ volume standard deviation of 8-13% among wild-type E15.5 embryos after normalising organ volumes by WEV. Developmental substage variability is prevalent in our control set because we sought to include as many wild-type specimens as possible in order to increase the power of our analyses. However, we have shown that we can control for developmental substage variability by adding a WEV term in our linear regression model. Without this substage correction, the statistical model can lead to false-positive results (Fig. 3A,B). WEV is a convenient staging metric as it can be easily calculated after embryo spatial normalisation, and is correlated with crown-rump length and whole-body weight, both previously used to stage embryos (Dagg, 1963; Peterka et al., 2002). Alternative methods that rely on the appearance of external features of the embryo (Theiler, 1989; Boehm et al., 2011; Geyer et al., 2017) may be more accurate, but these methods have yet to be automated for whole embryo 3D images. An alternative approach to automated staging involves spatially and temporally normalising embryos to a 4D (3D+time) dimensional population average, with developmental stage as the temporal dimension (Wong et al., 2015). This method was shown to provide high-resolution staging information and could identify developmental asynchrony across all organs. This 4D atlas was not used in this study as the latest developmental stage in the atlas, E14.0, does not overlap with our E14.5 dataset. The generation of a new 4D micro-CT atlas covering the E14.5 stage would require the breeding of a large number of embryos at various gestational timepoints, which is not currently possible. With our analysis, we identified 81 organs that show a statistically significant WEV effect (Table S2), which likely reflects different relative growth rates of various organs at different E14.5 developmental substages. This represents the most detailed embryo-wide data of E14.5 substage-specific organ growth rates that we are currently aware of.

Stratifying our wild-type specimens by sex enabled us to test the ability of LAMA to identify specific anatomical differences, as males and females are anatomically similar except for clear gonad differences. We found that we were able to uncover statistically significant gonad volume differences while keeping false positives low. To assess the performance on mutant data, we tested LAMA on two IMPC-generated knockout lines. The first (*Wfdc2^−/−^*) was predicted to display specific pulmonary abnormalities; the second (*Acan^−/−^*) to produce severely dysmorphic phenotypes across the whole embryo. Our automated analysis of E14.5 *Wfdc2^−/−^* embryos revealed two significantly smaller organ volumes: those of the trachea and bronchi, which are novel findings for this gene. These overlap broadly with the locations of the previously reported pulmonary-specific abnormalities in *Wfdc2*^−/−^ mice, including the absence of mature club cells from the bronchi and trachea, postnatally collapsed lung, reduced lung surfactant levels (Nakajima et al., 2019) and alveoli abnormalities (Zhang et al., 2020). The novel phenotypes reported here bring forward the time when gross abnormalities due to loss of *Wfdc2* first become visible during embryo development (previously postnatally), and therefore add new temporal information to the role of *Wfdc2* in pulmonary development. In addition, LAMA could recapitulate the majority of previously reported *Acan^−/−^* phenotypes, which will greatly speed up the annotation of this severely affected mutant. There were four significant organ volume differences for *Acan^−/−^* that have not previously been reported. It is possible that these are novel phenotypes, but it is difficult to confirm this by inspection of the micro-CT images alone. It is possible that the apparent abnormality is due to proximity to actual severe dysmorphology, which, during the registration process was warped along with the abnormal organ. For example, two of these organs without previous reports, sympathetic ganglia and the spinal cord marginal layer, are located close to the affected vertebra. Another reason for this discrepancy could be the different background strains used in this study and the previous studies.

As efforts are under way to reduce the numbers of animals used in scientific experiments, we wanted to test whether LAMA could identify dysmorphology with low mutant sample numbers. In addition, being able to use low sample numbers would let us investigate the effects of incomplete penetrance and variable expressivity, as well as providing phenotype data from mutant lines where many specimens do not reach the developmental stage being tested. To begin to answer this, in the sex difference test we show that increasing the control sample size from 10 to 49 greatly increases the power to detect anatomical differences, and that by using many controls, it is possible to sometimes uncover phenotype information even with a low mutant sample size of two. We also show that for six *Acan^−/−^* specimens, 42 specimen-level organ difference annotations were generated (Fig. 6A). Support for these specimen-level annotations comes from the fact that they mostly overlapped with the gene-level annotated organs (only 4/42 did not). These specimen-level annotations were strongly enriched for organs that had the largest volume difference relative to the control mean, which is expected due the reduced power of these tests being unable to detect smaller volume differences. There were differences between the annotations of the individual specimens, with specimens 15.1a and 9.2c lacking many of the bone annotations (Fig. 6A). These results show that, by using a large wild-type control sample number, useful phenotype information can be obtained when analysing low *N* mutant lines or even specimens individually.

Developmental delay is a common phenotype of knockout mice and such animals could pose a problem for this analysis as they could be smaller than the smallest mice in the baseline controls. In future, one way round this would be to include some E14.0 or E13.5 animals into our wild-type control set to extend the range to where we might expect to see developmental delay and improve the reliability of the model. In lieu of this, the whole embryo volume z-score (standard deviations from the wild-type mean) of each specimen, which is reported by LAMA, can be used to identify, and exclude, potentially delayed animals from analysis.

The choice of registration parameters can involve a compromise of balancing good registration accuracy on some organs with misregistration at others. We found that gonad registration, for example, could be improved by removing most of the registration constraints, but this led to over-warping at the heart. One solution to this could be to use multiple sets of registration parameters, each optimised to different parts of the atlas. Alternatively, approaches that directly segment organs without registration have been previously proposed (Yan et al., 2017; Ashish and Brusniak, 2018) but only on a limited number of organs, and these have yet to be applied to embryonic mice. LAMA is able to perform statistical analysis on the voxel intensities of the spatially normalised images, but we found that the image data used in this study contained large differences in intensity profiles that were possibly due to the different users and imaging equipment involved in image acquisition over a number of years. For this reason, we have concentrated our current analysis on organ volume differences and Jacobian determinant analysis, which are both more robust to varying intensity profiles. Future work will look towards employing more sophisticated image normalisation methods and exploring the use of other image features, such as textures, that may be less susceptible to intensity profile differences. Current work includes optimising registration parameters for E15.5 and E18.5 developmental timepoints (see Fig. S3 for current population average images), the latter being a key developmental time point for the analysis of gene mutations that result in perinatal lethality and subviability. Earlier stages, such as E12.5, may also be amenable to this analysis, but even earlier stages such as E9.5 may prove difficult for a registration-based approach due to the rapid developmental changes at this time point and extreme dysmorphology that is often caused by mutations that are lethal early in development. LAMA has been applied successfully to mouse bones that were dissected and scanned separately (N.R.H., unpublished) and can readily be adapted to other model systems where good registration between subjects is possible. This requires no software changes to the pipeline and only the registration parameters need to be optimised. Finally, we believe that the tools, resources and insights introduced in this article will accelerate the use of the rapidly increasing amounts of mouse embryo image data at the IMPC and within the wider mouse developmental biology community.

## MATERIALS AND METHODS

### Mice

All animals were housed and maintained in the Mary Lyon Centre at the MRC Harwell Institute under specific pathogen-free (SPF) conditions in individually ventilated cages adhering to environmental conditions, as outlined in the Home Office Code of Practice. All animal studies were licensed by the Home Office under the Animals (Scientific Procedures) Act 1986 Amendment Regulations 2012 (SI 4 2012/3039), UK, and additionally approved by the Institutional Ethical Review Committee. The Acan^tm1b^ allele was obtained by cre deletion of C57BL/6N-Acan^tm1a(EUCOMM)Hmgu^/H (EM:10224) mice as described previously (Birling et al., 2019 preprint). Homozygous mutants are named *Acan^−/−^* here. The C57BL/6NTac-Wfdc2^em1(IMPC)H^/H (EM:11407, homozygous mutants named *Wfdc2^−/−^* here) was obtained by genome editing as described previously (Mianné et al., 2017). Lines were maintained by crossing heterozygous animals with inbred C57BL/6N wild-type animals. Mice were euthanised by Home Office Schedule 1 methods.

### Micro-CT imaging of whole embryos

E14.5 female mice were sacrificed by cervical dislocation and the uterine horns removed into ice-cold phosphate-buffered saline (PBS). Embryos were extracted and a piece of yolk sac collected for genotype analysis. Embryos were fixed in 4% paraformaldehyde (PFA) at 4°C and left overnight. After fixation, the samples were washed and stored in PBS at 4°C. For staining, samples were rinsed in distilled H_2_O for 10 min before being submerged in 50% Lugol's solution and protected from light. Embryos were then left in the contrast agent for 2 days. Following staining, embryos were washed in distilled H_2_O for at least 1 h, embedded in 1% agarose (in distilled H_2_O) and left at room temperature for a minimum of 2 h.

High resolution micro-CT images (SkyScan 1172, Bruker) of agarose-embedded embryos were acquired at a source voltage of 70 kV, with the current set at maximum (∼100 mA). Specimens were imaged, in a standard orientation, at 3 μm with a 0.5 mm aluminium filter. X-ray projections were acquired at 0.25° increments, and reconstructed using the Feldkamp algorithm (Feldkamp et al., 1984) provided by NRecon (Bruker). Ring artefact corrections were applied as necessary. Reconstructions were automatically cropped to remove background and scaled to 14 μm isotropic voxels using the HARP software (Brown et al., 2018).

### Phenotyping pipeline implementation

The image registration pipeline was written in the Python programming language (Python 3.6+), adapting a modular design that allows for individual components (registration, inversion, statistics, etc.) to be run either sequentially or independently using simple TOML configuration files. Individual image registrations are performed using the elastix toolkit (Klein et al., 2010; Shamonin et al., 2014). The linear model analysis is implemented in R. All code is available on Github (https://github.com/mpi2/LAMA) and is tested to work on Ubuntu versions 18.04 and 20.4, as well as Windows 10. The use of interactive shell scripts that show how to use LAMA on a real dataset is described at https://github.com/mpi2/LAMA/wiki/walkthroughs. To make the installation of LAMA as easy as possible and to help data reproducibility, LAMA is available via the PyPi Python package repository (https://pypi.org/project/lama-phenotype-detection).

### Population average construction

Micro-CT images from 16 specimens of both sexes were used in the creation of the population average through a groupwise multi-level and multi-resolution registration process. For the first level, an initial fixed image was chosen at random and all other images were rigidly registered onto it. The registered images were averaged creating a rigidly aligned blurry average. This population average is invariant to the choice of the initial fixed image, because the composition of rigid transformations entails only a change of pose. For the second registration level, the rigid registration outputs were affinely registered onto the blurry average. A new (less) blurry average was computed from the outputs of this affine step. For the last registration levels, the process of alignment to the group average followed by recalculation of the average was repeated, using B-spline transformations, which allow local nonlinear deformations. We used a five-level Gaussian pyramid with increasing resolution for the images and five corresponding B-spline levels with decreasing control point spacing, with a final grid spacing of 8 voxels, to sequentially align coarser to finger anatomical structures (Fig. 1A). The parameter file for our population average and the final population average image available for download at https://www.doi.org/10.5281/zenodo.4559800.

### Image segmentation/E14.5 Atlas creation

Key anatomical structures within the E14.5 population average were identified manually by referencing the online digitised mouse atlas (Graham et al., 2015), which itself is based on *The Atlas of Mouse Development* (Kaufman, 1992). Structures that could be identified were restricted to those that showed good contrast and resolution within the population average. ITK-SNAP was used (Yushkevich et al., 2006; www.itksnap.org) to create the segmentations, using a variety of semi-automated and manual methods suited to the size and complexity of each of these anatomical structures, and combined into a single label file. These structures were then merged with some previous segmentations of brain structures derived from an E15.5 atlas (Wong et al., 2012) to give a total of 184 anatomical components. Small, spindly labels in the atlas were flagged by calculating a 3D euclidean distance transform for each label using the Python package edt (https://github.com/seung-lab/euclidean-distance-transform-3d) and flagging labels with a value <1.5. The resulting atlas and associated metadata files are available for download at https://www.doi.org/10.5281/zenodo.4559800.

### Generation of data for phenotype detection

Baseline and mutant specimens were registered onto the previously created population average image. The outputs of this registration include the rigid, similarity, affine and B-spline spatial transformations, co-registered images and the Jacobian determinants det(jac) of the composition of the B-spline transformations, a scalar field that describes the local volume change in each voxel. Statistical analysis of these outputs (described below) produces statistical parametric heat maps that can be overlaid onto the population average image or superimposed onto the input images by applying the inverse of the spatial transformations. The statistical parametric heat maps can be viewed with the Volume Phenotype Viewer (VPV) (Brown et al., 2018; https://github.com/mpi2/vpv).

### Statistical analysis

Multiple linear regression analysis was conducted in R (www.R-project.org) using the lm() function from the MASS package (Venables and Ripley, 2002). Benjamini-Hochberg FDR (BH-FDR) correction was carried out using the padjust R package (Benjamin and Hochberg, 1995).

### Voxel-level data

Jacobian determinants, generated at each voxel within the population average mask, provide information about how the registration has behaved locally. The scalar value of the Jacobian determinant at a given location is the factor by which that region has expanded [det(J_F_)>1] or shrunk [det(J_F_)<1] in volume during registration. This approach, known as tensor-based morphometry, can be used to reveal biologically significant localised shape or size changes within a population (Ashburner and Friston, 2000). To account for small registration inaccuracies, a Gaussian blur of full-width-half-maximum (FWHM) 100 μm is applied to voxel-level data. Each voxel is fitted to a linear model voxel∼genotype+WEV, where WEV stands for whole embryo volume. We use WEV as a proxy for developmental stage, and the addition of it as a fixed effect controls for changes that are due to developmental stage only. To account for multiple testing, the resulting *P*-value maps are corrected using the Benjamini Hochberg method. The final parametric heat maps are made by thresholding the t-static volume at q>0.05. This is output as a 3D image that can be overlaid onto the target or registered image in VPV.

### Whole-organ volume analysis

Organ volumes and whole-embryo volumes are derived for each specimen. Significant volume differences are detected by using an organ-specific linear model organ volume/WEV∼genotype+WEV. We apply a permutation-based approach (for each organ) for multiple testing correction described previously by Hrabě de Angelis et al. (2015). To summarise, organ-specific null distributions are generated by sampling synthetic mutants from the baseline data in such a way as to match the distribution of the number of mutant specimens per line. For example, if there are 40 mutant lines and 10 of these have *n*=3 and the other 10 have *n*=4, we create 50% synthetic lines with *n*=3 and 50% with *n*=4. Synthetic mutants and baseline controls are fitted to the linear model as described above and the genotype effect *P*-values are computed. Alternative distributions are made by computing genotype effect *P*-values from testing the real mutants of each line. To obtain a dataset-wide *P*-value threshold per organ, combined null and alternative *P*-values for the organ are ranked and a descending *P*-value threshold search is conducted starting at *P*=0.05 until a threshold is found where the proportion of alternative *P*-values under the threshold divided by the proportion of null *P*-values under the threshold is <0.05. Mutant *P*-values below this threshold are assigned as significant, which sets the organ-specific FDR to 5%.

### Detection of sex-specific differences

For the initial experiment that tested for a sex effect on organ volume (Fig. 4A,B), organ volumes from all available males and females were fitted to the linear model organ volume/WEV∼sex+WEV. False discovery correction was performed across all organs using the Benjamini Hochberg method, as it was not possible to permute the data as all the data was fitted to the model. For the organ volume analysis that tested varying sample numbers (Fig. 4C,D) organ-specific *P*-value thresholds were generated using the permutation based method described above. Only experiments where combinations of male and female sample size allowed at least 500 unique null permutations were included. Jacobian determinant analysis (Fig. 4E,F) was carried out as described above.

### Variable penetrance and low *N*

To identify potentially variable expressivity or incomplete penetrance of organ volume phenotypes, LAMA routinely performs what we term ‘specimen-level analysis’. After the analysis of a mutant line as a group, each individual specimen is analysed singly, i.e. the organ volume, or voxel value, for a single specimen is fitted to a linear model along with baseline controls to obtain a specimen-level *P*-value for the genotype effect. Owing to the reduction in power from using one specimen, we have set the FDR threshold for the specimen-level analysis to 20%. The voxel-level data are processed similarly to the gene-level voxel data and the voxels are thresholded to an FDR of 5%.

### Optimisation and quality control

At each level and resolution of the registration process, the similarity metric output by elastix is plotted against iteration number, allowing the user to visually decide when the optimisation process has converged, and set an optimal number of iterations in future.

Image registration can sometimes fail to produce acceptable results, e.g. when the moving image is over fitted to the fixed image, producing unrealistic warping. To check for issues such as these, after each registration stage an additional HTML report was generated that contains mid-sagittal slices for each registered image for rapid quality control. Another issue that can be encountered using B-spline-based image registration is folding of the deformation field, which prevents topology preservation and has no inverse transformation. This problem can be detected by the presence of negative Jacobian determinants. In that case, only pixels with negative Jacobian determinants are displayed, allowing the user to quickly identify problematic regions within the registered images. The registration stage of the analysis is the most time-consuming part of the pipeline, and so it is a requirement to be able to optimise registration parameters, especially within a high-throughput context.

### Comparing *Acan* phenotypes found by LAMA to known phenotypes

Tables of known phenotypes were generated by querying MGI phenotype pages for the gene of interest *Acan:* www.informatics.jax.org/marker/phenotypes/MGI:99602*.* Only phenotypes generated from homozygous null strains were included to aid in the comparison. Duplicate and redundant phenotypes (e.g. abnormal bone structure if more specific bone phenotypes were present) were removed. Phenotypes that might not translate to a gross anatomical dysmorphology that could be potentially detected by LAMA (e.g. deafness) were also removed.

## Supplementary Material

Reviewer comments
