# Supplementary material for: LAMA: automated image analysis for the developmental phenotyping of mouse embryos
Source: Development. 2021 Mar 24;148(18):dev192955. doi: 10.1242/dev.192955 (PMC8015254; doi:10.1242/dev.192955)
Supplement: Reviewer comments [file develop-148-192955_review_history.pdf]

LAMA: automated image analysis for the developmental phenotyping of mouse embryos

Neil R. Horner, Shanmugasundaram Venkataraman, Ramón Casero, James M. Brown, Michael D. Wong, Matthijs C. van Eede, R. Mark Henkelman, Sara Johnson, Lydia Teboul, Sara Wells, Steve D. Brown, Henrik Westerberg and Ann-Marie Mallon
DOI: 10.1242/dev.192955

Editor: Liz Robertson

Review timeline
Original submission: 19 May 2020
Editorial decision: 20 August 2020
First revision received: 19 November 2020
Accepted: 21 December 2020

Original submission

First decision letter

MS ID#: DEVELOP/2020/192955

MS TITLE: LAMA: Automated image analysis for developmental phenotyping of mouse embryos

AUTHORS: Neil R Horner, Shanmugasundaram Venkataraman, Ramon Casero, James M Brown, Sara Johnson, Lydia Teboul, Sara Wells, Steve D M Brown, Henrik Westerberg, and Ann-Marie Mallon

First let me apologise on behalf of Development for the prolonger delay in getting back to you.

I have now received all the referees' reports on the above manuscript, and have reached a decision. The referees' comments are appended below, or you can access them online: please go to BenchPress and click on the 'Manuscripts with Decisions' queue in the Author Area.

As you will see, the referees express considerable interest in your work, but have some significant criticisms and recommend a revision of your manuscript before we can consider publication. Reviewer 2 details some very specific issues that we hope you can address in making the revisions.

Regarding the concerns expressed by the referee at MICe (this was Mark Henkelman; he is aware that I am sharing his identity with you), it is in general very hard for us as a journal to be prescriptive about authorship allocation, since we aren't really in a position to know who contributed what to a paper. We always recommend that authors (and potential authors) discuss in detail before submitting a paper, and it's a shame that this doesn't seem to have happened in this case. We have discussed the situation with an advisor who is familiar with the tools and the community; both we and they think there may be a case for offering authorship to the relevant MICe contributors – in that their input was influential in the early stages of the project, but I'm not sure that we would insist on it if you disagree strongly. We would much prefer that you could work this out and come to an agreement with Dr Henkelman and his colleagues would therefore ask you to get in touch and discuss the situation in an open and collaborative manner – and I'm confident that Dr Henkelman would be open to this kind of discussion. We'd also ask you to keep us updated with the outcome of these discussions, particularly if you're not able to reach an amicable arrangement.

© 2021. Published by The Company of Biologists under the terms of the Creative Commons Attribution License (https://creativecommons.org/licenses/by/4.0/).

If you are able to revise the manuscript along the lines suggested, I will be happy receive a revised version of the manuscript. Your revised paper will be re-reviewed by one or more of the original referees, and acceptance of your manuscript will depend on your addressing satisfactorily the reviewers' major concerns. Please also note that Development will normally permit only one round of major revision.

We are aware that you may currently be unable to access the lab to undertake experimental revisions. If it would be helpful, we encourage you to contact us to discuss your revision in greater detail. Please send us a point-by-point response indicating where you are able to address concerns raised (either experimentally or by changes to the text) and where you will not be able to do so within the normal timeframe of a revision. We will then provide further guidance. Please also note that we are happy to extend revision timeframes as necessary.

Please attend to all of the reviewers' comments and ensure that you clearly highlight all changes made in the revised manuscript. Please avoid using 'Tracked changes' in Word files as these are lost in PDF conversion. I should be grateful if you would also provide a point-by-point response detailing how you have dealt with the points raised by the reviewers in the 'Response to Reviewers' box. If you do not agree with any of their criticisms or suggestions please explain clearly why this is so.

Reviewer 1

Advance summary and potential significance to field

Unclear.

Comments for the author

Major repositioning needed

Reviewer 2

Advance summary and potential significance to field

The authors presented in this manuscript an image pre-processing application Lightweight Analysis of Morphological Abnormalities (LAMA) that performs image pre-processing, registration, statistical annotation and image segmentation of 3D volume data acquired from microCT. The authors demonstrated that by using LAMA, a detail E14.5 mouse embryo atlas is generated, and it can be used to distinguish the dataset based on gender, as well as identified developmental abnormalities in mutant embryos, as demonstrated by using Wfdc2 and Acan as examples. Although the reviewer thinks this manuscript has the merits to be published in the Journal of Development, a Major Revision is recommended based on several considerations listed below.

1. Be more concise for the introduction and address the advancement of the application LAMA compared to previous limitation. In addition to an easy to be distributed Python package, how does it advance the field in terms of processing speed, application to different embryonic stages or other model system. Is it only for E14.5 embryo?
2. Regenerate Figures and Tables as some are hard to read the legend (ex. Figure 1E) or too small compared to other panels (ex. Figure 5F, Figure 6 A-B). The authors should put in efforts to make the Figures and Tables to best represent the results and easy to follow for the readers.
3. The authors have done an extensive amount of analysis, however, some of them were not explained in detail in the result section. For example, in line 301 "(see fig 6C-D for examples of…)", the detail should be include in the result section. Also, a lot of information described in the methods section should be include in the result section to help readers better understand and intepretate the results (ex. whole organ volume developmental substage analysis and detection of sex-specific differences).
4. In the discussion line 323-324, the authors stated that "(and will be adapted for other stages)". So, what is the current limitation for other IMPC centers to adopt LAMA for E15.5 and E18.5 embryo analysis? Can researchers adopt it for other developmental stages easily or further development is required? This should be clearly stated to differentiate what have been achieved in

© 2021. Published by The Company of Biologists under the terms of the Creative Commons Attribution License (https://creativecommons.org/licenses/by/4.0/).

this manuscript and what needs to be further developed in the future study. It is not clearly stated in the manuscript.

Comments for the author

Comments 1. in line 50 "approximately one third of all knockout mouse lines exhibit embryonic, or early postnatal lethality or subviability". Please be more specific about whether it is the data from IMPC only, or from "all" of the knockout mouse lines that are ever being generated.
2. In paragraph 124 - 136: It will be easier for the readers if you summarize the issues that LAMA can address to highlight the novelty/advantages of the software to get the concept of the paper. The rest of the paragraph reads more like result/discussion instead of introduction. Because after reading this paragraph, it is still not clear to me what LAMA can do compare to what has been developed before.
3. What are the differences when compare LAMA to previous described methods for E15.5 registration?
4. Be more specific on the result title "overview of the phenotyping pipeline for E14.5 mouse embryos". Is it the IMPC pipeline or LAMA analysis pipeline?
5. Line 188 - 189: what is the standard deviation of the CRL for the 16 embryos?
6. The text in figure 2E are not readable. A figure legend listing which part is labeling in figure 2B will be extremely beneficial for highlighting the result.
7. Table 1 should be reproduced in a more readable, organized format, maybe a spreadsheet as a supplement instead.
8. Line 211 - 215: the rational for selecting the smallest/biggest as mutants is not so clear. The authors described the E14.5 "substage" based on WEV, and 8 with the lowest and 8 with the highest were selected.
Since there's 99 in total, maybe selecting the groups that are 2 SD below or over the average for comparison will be more beneficial? Also, have the average WEV of E13.5 and E15.5 been determined? The lower end of the WEV might fall into more at E13.5 due to developmental delay in some null embryo cases. It will enhance the significance of the software capability if the authors can demonstrate that LAMA can clearly distinguish the embryos with growth retardation. Also, as authors were trying to account for the substage effects to minimize the false positive results, how close the substages is to E13.5 or E15.5 should also be considered. In the other word, if a group of E13.5 or E15.5 embryos were put into analysis, would it be successfully identified?
9. Line 206 - 263: The authors states here "...even with a low mutant sample size". Based on the experiment described in this section by labeled the female as "mutant", it compares 49 males and 8 females. Can it still detect the mutants if the sample size is lower than 8? As the authors stated in line 102 - 104 "...often have mutant sample sizes that are significantly lower than the desired eight mutant..." can the same result be produced if 3 to 4 female + 49 males were analyzed? Can the same analysis be done on male?
10. What is the secondary viability calls for Wfdc2 and Acan?
11. It is confusing on how the authors define the difference between "gene-level" and "specimen-level". Is it referring to gene expression or grouped versus individual mutant embryo?
Minor comments:
- Font selection or pdf file conversion: it seems like there's always an extra space after the alphabet "w" in each word, makes it hard to read the manuscript. Please check the pdf conversion.
- Line 41: "phenotyping procedures": capital P at the beginning of the sentence.
- Line 98: add "." At the end of the sentence "...eight mutants and controls"
- Line 205 - 207: reword this sentence.
- Line 102 - 104: "often have mutant sample sizes that are significantly lower that the desired eight mutant..."
should be lower than the desired.
- Line 269: add "." After "and other project"
- Paragraph from line 93 to 107 read more like discussion instead of introduction.
- Paragraph 117 - 122: when you referring to the uncertainty around the exact time of conception, are you referring to it as off by several hours or by a day? If wild type littermates are selected, as well as proper time-mating for breading, then off by a day shouldn't be an issue. Are you trying to refer to E14.5 the embryos are still going through a very rapid organogenesis (TS21 ? TS22)?
- Line 132 - 135: This should be two sentences "...modalities. And to aid this..."

© 2021. Published by The Company of Biologists under the terms of the Creative Commons Attribution License (https://creativecommons.org/licenses/by/4.0/).

First revision

Author response to reviewers' comments

We would like to thank the reviewers for their reviews on the submission draft especially during these challenging times. Overall we largely agree with the comments and have sought to address all of them positively within this resubmission.

We now address the comments in turn:

Reviewer 2
C1: Be more concise for the introduction and address the advancement of the application LAMA compared to previous limitation. In addition to an easy to be distributed Python package, how does it advance the field in terms of processing speed, application to different embryonic stages or other model system. Is it only for E14.5 embryo?

We have made the introduction more concise making it more readable, and focus on the key differences. The main advancements of LAMA over previous attempts are now more clearly stated, namely an improved registration strategy leads to a gain in speed by reducing the total number of registrations required for each specimen. As we can reuse specimens for each mutant line, the total number of specimens can be dramatically increased. This gives us increased statistical power, and allows us to test for dysmorphology in individual specimens, which is very important as it allows us to gain phenotype information with lines with *N*=1 or lines with highly variable penetrance/expressivity. Our novel E14.5 atlas we present is also a big improvement, allowing us to automatically assign anatomical phenotypes for this developmental stage, which was not previously possible. We are currently adapting LAMA to both E15.5 and E18.5 stages and include this in the discussion (along with images of the latest population averages for this stage Fig. S3). We have had some success applying LAMA to analysis of bones (unpublished) and have also mentioned this in the discussion.

C2: Regenerate Figures and Tables as some are hard to read the legend (ex. Figure 1E) or too small compared to other panels (ex. Figure 5F, Figure 6 A-B). The authors should put in efforts to make the Figures and Tables to best represent the results and easy to follow for the readers.

The figures presented for the initial submission were format-free in accordance with Development's author guidelines. For this full resubmission, we have updated all the figures, including increasing font size, reducing white space, and moving some elements to supplementary figures in order to allow increasing size of panels for easier viewing. The tables of atlas label size and significant labels from the sex effect study have been moved to supplementary excel files as they contain a fair amount of data, which is more suited to spreadsheet viewing. Some of the specimen vignettes have been altered, for example Fig. 5 is now displaying coronal and sagittal views to better display the affected organs, but the specimens used remain the same.

C3: **The authors have done an extensive amount of analysis, however, some of them were not explained in detail in the result section. For example, in line 301 "(see fig 6C-D for examples of…)", the detail should be include in the result section. Also, a lot of information described in the methods section should be include in the result section to help readers better understand and intepretate the results (ex. whole organ volume, developmental substage analysis and detection of sex-specific differences).**

Some of the detail from the Methods section "Whole organ volume analysis" has been moved to the results section "Overview of the LAMA phenotyping pipeline" sentence beginning "To account for overall size differences..".

The details of the linear models used for each experiment in the results section: "Developmental substage" are now included. The linear model notation has also been updated to show the normalisation of organ volumes as it might have been unclear previously. The corresponding section in the methods has been removed as it has become redundant. Please note, the number of specimens used is 93 as stated in the results and not 99 that was previously stated in the methods.

© 2021. Published by The Company of Biologists under the terms of the Creative Commons Attribution License (https://creativecommons.org/licenses/by/4.0/).

Detection of sex-specific differences Methods section.
Some of the details in this section repeated much of the results section: “Optimal sample size for phenodeviance testing”. This has been removed leaving only some experimental setup that we think are too detailed for the results section. The results section has been partly reworded to hopefully make more clear the details of the experiment.

C4: .In the discussion line 323-324, the authors stated that “(and will be adapted for other stages)”. So, what is the current limitation for other IMPC centers to adopt LAMA for E15.5 and E18.5 embryo analysis? Can researchers adopt it for other developmental stages easily or further development is required? This should be clearly stated to differentiate what have been achieved in this manuscript and what needs to be further developed in the future study. It is not clearly stated in the manuscript.

We have further clarified the situation regarding other stages and other model systems within the discussion. No other software developments are required for LAMA to operate at other stages or for other model systems. We have included an additional figure of our current LAMA-generated averages at E15.5 and E18.5 data from IMPC (Fig S3) that were generated with LAMA after optimising the registration parameters. We are also drafting a protocols paper, in order to, help new users with this process.

Major Comments:
C1:in line 50 "approximately one third of all knockout mouse lines exhibit embryonic, or early postnatal, lethality or subviability". Please be more specific about whether it is the data from IMPC only, or from "all" of the knockout mouse lines that are ever being generated.

This sentence has been updated to explicitly name IMPC and EUMODIC as the source of the lethality data rate.

C2: It will be easier for the readers if you summarize the issues that LAMA can address to highlight the novelty/advantages of the software to get the concept of the paper. The rest of the paragraph reads more like result/discussion instead of introduction. Because after reading this paragraph, it is still not clear to me what LAMA can do compare to what has been developed before.

This is a similar comment to Reviewer 1’s comment 1. We have reworked the introduction to put more attention on what is novel about LAMA. We have stressed that it is possible to run many more baselines than previous mouse phenotyping tools, due to the lack of groupwise registration steps all the specimens being in the same coordinate space, we can reuse baseline registered data across all mutants. So we have increased the baseline control number from 8 to 93. This allows us to increase our power, which allows for the analysis of mutant lines with low n (as low as 1). This means we can analyse individual specimens, and KO lines with low sample numbers, which has not previously been possible.

C3: What are the differences when compare LAMA to previous described methods for E15.5 registration?

The differences have been further clarified in both the introduction and discussion (See C2).

C4 Be more specific on the result title "overview of the phenotyping pipeline for E14.5 mouse embryos". Is it the IMPC pipeline or LAMA analysis pipeline?

The section titled "overview of the phenotyping pipeline for E14.5 mouse embryos" has been changed to "Overview of the LAMA phenotyping pipeline". This makes it clear that we are referring to LAMA and not the IMPC embryo pipeline. And the reference to E14.5 has been removed as it is not specific to a developmental stage.

C5 Line 188 - 189: what is the standard deviation of the CRL for the 16 embryos?

The standard deviation of the average embryo inputs (0.52mm) has been added to the result section.

© 2021. Published by The Company of Biologists under the terms of the Creative Commons Attribution License (https://creativecommons.org/licenses/by/4.0/).

C6 The text in figure 2E are not readable. A figure legend listing which part is labeling in figure 2B will be extremely beneficial for highlighting the result.

Fig. 2 (the atlas figure) has been updated with label numbers highlighting the visible labels. The organ names can be looked up in Table S1 excel sheet. Full label names would not fit on the image and we chose to refer readers to the excel file rather than make a very large legend for the figure (but this can easily be added if the review deems it necessary). An image inset has been added focusing on a region of complex labelling. The plot showing the distribution of organ volume sizes was removed as it provided little information. We also provide a movie (Movie 1) in the current submission, which shows a rotating 3D rendered atlas that gives a good indication of the amount of detail that exists in the atlas. The font size has also been increased.

C7 Table 1 should be reproduced in a more readable, organized format, maybe a spreadsheet as a supplement instead.

Table 1 is now Table S1 in excel format.

C8 Line 211 - 215: the rational for selecting the smallest/biggest as mutants is not so clear. The authors described the E14.5 "substage" based on WEV, and 8 with the lowest and 8 with the highest were selected. Since there's 99 in total, maybe selecting the groups that are 2 SD[1] below or over the average for comparison will be more beneficial? Also, have the average WEV of E13.5 and E15.5 been determined? The lower end of the WEV might fall into more at E13.5 due to developmental delay in some null embryo cases. It will enhance the significance of the software capability if the authors can demonstrate that LAMA can clearly distinguish the embryos with growth retardation. Also, as authors were trying to account for the substage effects to minimize the false positive results, how close the substages is to E13.5 or E15.5 should also be considered. In the other word, if a group of E13.5 or E15.5 embryos were put into analysis, would it be successfully identified?

Our initial intention was to use a standard deviation range to select for our large and small group, as the reviewer suggested. However the mean SD of the large group (1.9) and small group (-1.7) was as near to 2SD as we could get with the available data. The SD of the two groups is now reported in the results section. Please note, the number of specimens used is 93 as stated in the results and not 99 that was previously stated in the methods. This was due to the removal of QC issue specimens without the text being updated accordingly.

The most challenging comment for us is the question around developmental delay and unfortunately we do not have E13.5 or E15.5 wild-type embryos imaged at Harwell following the same staining protocol. And we know from experience that using data with different staining protocols will lead to spurious results. Within the current constraints due to the pandemic, as well as ethical considerations, this would delay resubmission by many months.

As the gestational age of E14.5 embryos spans several hours and we use a large amount of baselines, any KO embryos that are delayed by possibly several hours will be within the WEV range of our baselines and so will register correctly to our population average (everything else being the same). However, if the mutants are outside of the baseline WEV range (+- 2.0 sd) then comparison to our wild type set may not be informative and could potentially introduce spurious results. With your comments in mind we have now implemented a whole embryo volume sd check, which flags the embryo as being potentially developmentally delayed or as being too old. It is then up to the user as to how to interpret this given the rest of the data

C9 Line 206 - 263: The authors states here "...even with a low mutant sample size". Based on the experiment described in this section by labeled the female as "mutant", it compares 49 males and 8 females. Can it still detect the mutants if the sample size is lower than 8?

The results section: “Optimal sample size for phenodeviance testing” describes a series of experiments where different numbers of male and female specimens are used in order to test the effect of using different numbers of baseline and mutants. Fig. 4C shows the results of each of these experiments, and it can be seen from this plot that using a female n=2 significant organ differences

© 2021. Published by The Company of Biologists under the terms of the Creative Commons Attribution License (https://creativecommons.org/licenses/by/4.0/).

in the gonad are observed. We do not go as low as n=1 in this experiment as there are not enough baselines to generate enough permutations (because we have split the dataset between males and females). When testing the two mutant lines in the later sections, we are able to use a mutant sample size of 1 as we are able to use all 93 baselines. The following sentence in the results section has been included to describe more clearly the aim and setup of the experiment

“We next wanted to address the effect of sample size on the phenodeviance detection sensitivity (in this case the ability to differentiate between male and female gonads and lenses). To do this, the previous experiment was repeated, but with varying numbers of males or females specimens, in this way replicating the effect of testing mutant lines containing various sample numbers and with different baseline control sample numbers (ranging in from 2-8 females and 10-49 males).”

C10 **What is the secondary viability calls for Wfdc2 and Acan?**

Secondary viability data has been added to the Wfdc2 and Acan results sections. Wfdc2 are viable at E18.5, but have insufficient numbers at earlier stages to make a call. Acan mutants are viable at E12.5, E14.5 and E18.5

C11 **It is confusing on how the authors define the difference between "gene-level" and "specimen-level". Is it referring to gene expression or grouped versus individual mutant embryo?**

Gene-level refers to the phenotype uncovered when analysing mutant specimens with the same deletion as a group. Specimen-level refers to the phenotype of individual specimens (using a mutant sample size of 1). This has been clarified in the final sentences of the “Overview of the LAMA phenotyping pipeline” section. The terms are also explained in the “Automated identification of developmental phenotypes in E14.5 mice embryos” section upon first use. We hope this makes the usage of these terms clear.

Minor comments

1.
The PDF conversion was poor. This has been fixed.

2.
The capital P is not visible for us at Line 41.

3.
The sentence at Line 205 - 207
"Embryos harvested at E14.5 represent a range of developmental substages(DSS) and have rapidly developing anatomy and so it is crucial for a high throughput data analysis pipeline to account for this variation in the data"

has been reworded to:
“As the developmental substage of E14.5 embryos has been shown to be an important consideration when manually phenotyping embryos (Geyer et al., 2017), we next performed a series of experiments to gauge the effect that developmental stage has on our automated phenotyping results.”

4.
Fixed typo

5.
- Paragraph from line 93 to 107 read more like discussion instead of introduction. This part of the introduction has been rewritten.

6.
- Paragraph 117 - 122: when you referring to the uncertainty around the exact time of conception, are you referring to it as off by several hours or by a day? If wild type littermates are selected, as well as proper time-mating for breading, then off by a day shouldn't be an issue. Are you trying to

© 2021. Published by The Company of Biologists under the terms of the Creative Commons Attribution License (https://creativecommons.org/licenses/by/4.0/).

refer to E14.5 the embryos are still going through a very rapid organogenesis (TS21 ? TS22)?

The uncertainty of gestational age will be several hours not days, which we have now stated. We have added text and reference to articles indicating that there is significant intra-litter, differences in stages of embryos. Also, if using littermate controls only, we would not have enough statistical power to identify morphological differences as we usually only get one or two WT littermate embryos scanned for each mutant line.

Even though organogenesis is largely complete at E14.5, there are morphological differences between the embryos that are dependent on developmental substage of the embryos. For example cleft palate, and the stage of ventricular septum closure is dependent on E14.5 sub stage (Geyer et. al. 2017)

7.
This paragraph has been rewritten.

Second decision letter

MS ID#: DEVELOP/2020/192955

MS TITLE: LAMA: Automated image analysis for developmental phenotyping of mouse embryos

AUTHORS: Neil R Horner, Shanmugasundaram Venkataraman, Ramon Casero, James M Brown, Michael D Wong, Matthijs C van Eede, R Mark Henkelman, Sara Johnson, Lydia Teboul, Sara Wells, Steve D M Brown, Henrik Westerberg, and Ann-Marie Mallon
ARTICLE TYPE: Techniques and Resources Article

I am happy to tell you that your manuscript has been accepted for publication in Development, pending our standard ethics checks.

Reviewer 2

Advance summary and potential significance to field

The authors presented in this manuscript an image processing application Lightweight Analysis of Morphological Abnormalities (LAMA) that performs image pre-processing, registration, statistical annotation and image segmentation of 3D volume data acquired from microCT. The authors demonstrated that by using LAMA, a detail E14.5 mouse embryo atlas is generated, and it can be used to distinguish the dataset based on gender, as well as identified developmental abnormalities in mutant embryos, as demonstrated by using Wfdc2 and Acan as examples to identify organ differences at specimen level. With the advancement of registering all the specimens into the same coordinate compared to previous developed method, this paper shows the improvement of not only LAMA can identify the organ-specific difference at a grouped/gene level but also at individual specimen level to account for penetrance issues. This tool will greatly improve the ability to identify potential phenotypes from the high-throughput IMPC pipeline, as well as beneficial to researchers who want to utilize microCT imaging for mouse phenotyping.

Comments for the author

The authors have addressed all of my comments from the previous review and there's no major comment for this revision.

Minor comments:
-deformable Jacobians: there's a space in all the bold text between f and o.

© 2021. Published by The Company of Biologists under the terms of the Creative Commons Attribution License (https://creativecommons.org/licenses/by/4.0/).

Reviewer 3

Advance summary and potential significance to field

This paper reports an automated image registration and phenotype identification pipeline for the high throughput analysis of mutant mouse embryos. The specific advance here over other reports is the pairwise comparison strategy aimed at identifying phenotype differences using small sample numbers.

Comments for the author

The authors have prepared a strong response to previous reviewer comments and the study appears complete.

© 2021. Published by The Company of Biologists under the terms of the Creative Commons Attribution License (https://creativecommons.org/licenses/by/4.0/).
